# Exploring spatial and temporal trends in the soundscape of an ecologically significant embayment

**DOI:** 10.1038/s41598-017-06347-0

**Published:** 2017-07-18

**Authors:** R. L. Putland, R. Constantine, C. A. Radford

**Affiliations:** 1 Leigh Marine Laboratory, Institute of Marine Science, University of Auckland, PO Box 349, Warkworth, 0941 New Zealand; 2 School of Biological Sciences, University of Auckland, Private Bag 92019, Auckland, 1142 New Zealand

## Abstract

The Hauraki Gulf, a shallow embayment in north-eastern New Zealand, provides an interesting environment for ecological soundscape research. It is situated on a tectonic plate boundary, contains one of the busiest ports in the southern hemisphere and is home to a diverse range of soniferous animals. The underwater soundscape was monitored for spatial and temporal trends at six different listening stations using passive acoustic recorders. The RMS sound pressure level of ambient sound (50–24,000 Hz) at the six listening stations was similar, ranging from 90–110 dB re 1 μPa throughout the recording period. Biophony had distinct temporal patterns and biological choruses of urchins were significantly correlated to temperature. Geophony and biophony followed the acoustic niche hypothesis, where each sound exhibited both temporal and frequency partitioning. Vessel passage sound were identified in 1.9–35.2% of recordings from the different listening stations. Vessel sound recorded in the Hauraki Gulf has the potential to mask concurrent geophony and biophony, sounds that may be important to marine life. This study provides a baseline of ambient sound, useful for future management strategies in shallow embayments where anthropogenic pressure is likewise increasing.

## Introduction

Sound emanates in the ocean from a myriad of sources, including geophysical and meteorological events (geophony), biological activity or vocalisations (biophony) and anthropogenic activities (anthropophony). The combination of these sounds in an environment is referred to as the soundscape^1^.

The contribution of geophony to the soundscape is intense, but highly variable. In the nearshore environment, it is mostly caused by surface agitation from wind-generated waves^2, 3^ and rain^4^, producing sound in the mid (200–2,000 Hz^3^) and high (15–20 kHz^2^) frequencies, respectively. In contrast, in the deep ocean geophysical activity, such as earthquakes and volcanic eruptions are the main contributors to the low frequency spectrum (<100 Hz^5^).

Four major groups of marine animals are known to produce sound and contribute to the biophony, crustaceans^6^, urchins^7^, fish^8^ and marine mammals^9^, encompassing a wide frequency range from 10 Hz to over 20 kHz. Various crustaceans produce mechanical sounds, snapping shrimp (*Synalpheus* sp.) when feeding^10^, spiny lobster (*Panulirus interruptus*) when interacting with potential predators^11^ and paddle crabs (*Ovalipes trimaculatus*) while establishing territories and/or attracting mates^12^. Other invertebrates such as urchins and mussels only produce incidental sounds whilst feeding^7^. Fish typically produce sound for social cohesion^13^, reproductive displays and territorial defence^8^. Similarly, marine mammals use sound as a primary means of communication and toothed whales have developed sophisticated echolocation systems to find prey^9^.

Biophony, unlike geophony, provides regular contributions to the soundscape^2^ with daily, monthly and seasonal trends identified from both temperate and tropical systems^14–16^. For example, the crepuscular activity of urchins and some fish causes a marked increase in ambient sound level more commonly known as the dawn and dusk chorus^3, 7, 14^. Over the course of a lunar cycle, reef sounds will also vary significantly, with more intense sounds produced during the new moon and less intense sounds during the full moon^16^. Furthermore, ambient sound levels have been observed to be higher during summer than winter in temperate coastal habitats^2, 15^. In the open ocean seasonal sound intensity is often dependent on marine mammal species, for example humpback whales (*Megaptera novaeangliae*) vocalise during their winter-spring breeding season, significantly increasing ambient sound levels at low to mid-frequencies (100–2,000 Hz)^17^.

Geophony and biophony have been present in the world’s oceans for thousands of years. However, in the last half a century^18^, there has been increasing inputs of sound into the world’s oceans by human activity, such as from commercial shipping, fishing and aquaculture, dredging, geophysical surveying, oil drilling and sonar systems^19^. Distant shipping is the principal source of sound between 50 and 500 Hz^5^ and is ubiquitous across the world’s oceans. Critically, the general increase of sound in the ocean may be causing homogenisation or fragmentation of the soundscape potentially threatening marine organisms that make use of sound for everyday life.

Soundscapes are not static, they change according to both spatial and temporal patterns. Past studies have mostly focused on analysing soundscapes by using short-term measurements at many locations and habitats^20–24^. Alternatively, soundscape ecologists have used long-term measurements to focus on a single species^25, 26^ or comprehensively understand temporal patterns at a specific location^2, 16, 27, 28^. Such studies have proved vital in improving knowledge of soundscape ecology. However, future investigation needs to combine all aforementioned (a long time-frame, different habitats and locations as well as a high sampling rate) to determine or confirm acoustic patterns and ecological processes. The goals of this research are to describe the soundscapes in an ecologically important embayment over varying spatial and temporal scales, and to quantify sources of the ambient soundscape including geophony, biophony and anthropophony.

## Results

**Figure 1:**
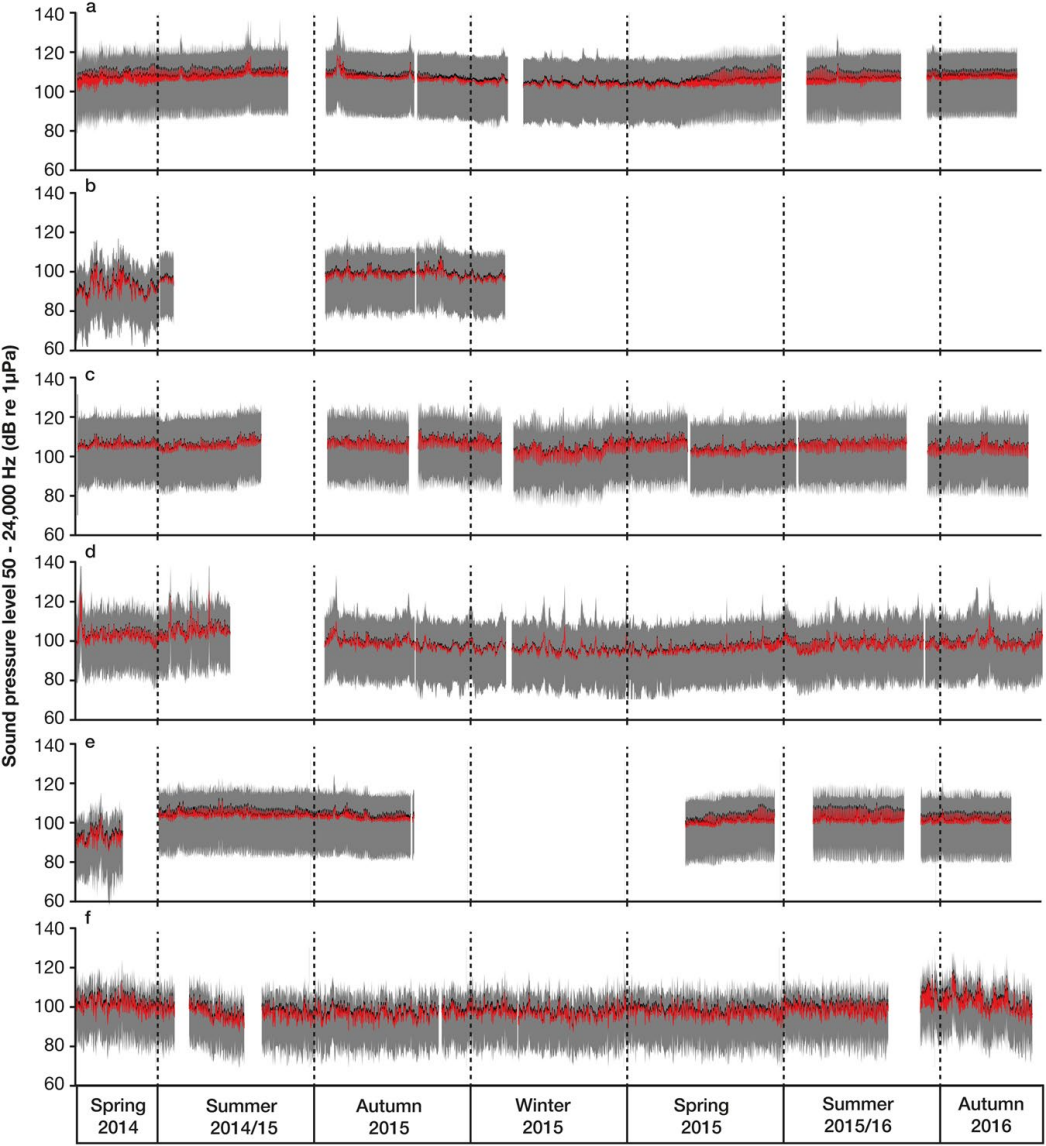
RMS level (red line), median (black line), 5^th^ and 95^th^ percentile (shaded area) of broadband sound pressure level between 50–24,000 Hz for daily (day, dawn, dusk and night) time categories over the duration of deployment for each of the six listening stations, (**a**) Horn Rock, (**b**) Waiheke Island, (**c**) Bean Rock, (**d**) Shearer Rock, (**e**) Flat Rock, and (**f**) Jellicoe Channel.

Broadband root-mean-squared (RMS) and median sound pressure levels (SPL) (50–24,000 Hz) were similar for all listening stations, ranging from 90–110 dB re 1 µPa (Fig. 1, Supplementary Fig. S1) for the entire recording period. Season and time of day significantly affected RMS SPL (three way ANOVA, F_3_ = 3434 p < 0.001 and F_3_ = 2768 p < 0.001). RMS SPLs for dusk recordings were significantly higher than other times of day at Horn Rock, Shearer Rock, Flat Rock and Jellicoe Channel. RMS SPLs during summer were also significantly higher than winter levels at Horn Rock, Shearer Rock and Jellicoe Channel. At Horn Rock (Fig. 1a), fluctuations in the RMS and median SPLs during the spring and summer suggested the presence of a dominant sound source at dusk. Whereas, at Bean Rock RMS SPLs (Fig. 1c) were significantly higher during the day than night and there was no significant seasonal variation, whilst at Waiheke Island (Fig. 1b) there was a significant difference in RMS SPL in spring versus autumn for all times of day. Large spikes in the SPLs were recorded occasionally at Horn Rock and Shearer Rock (Fig. 1a,d), which was attributed to anthropophony of the intensive fishing activity that occurs at these two sites. Overall, the range of 5^th^ and 95^th^ percentile SPLs throughout the deployment period was 75–125 dB re 1 µPa for all listening stations (Fig. 1, Supplementary Fig. S1). At Flat Rock there was a clear change in SPL between spring 2014 and the other deployment periods (Fig. 1e), due to the repositioning of the listening station because of the failure of the acoustic release. Percentile analysis shows that Jellicoe Channel was the quietest site because the 95^th^ percentile of the SPL was significantly lower than the median (Fig. 1f), which shows that this site is effected by loud transient sound, such as vessels.

**Figure 2:**
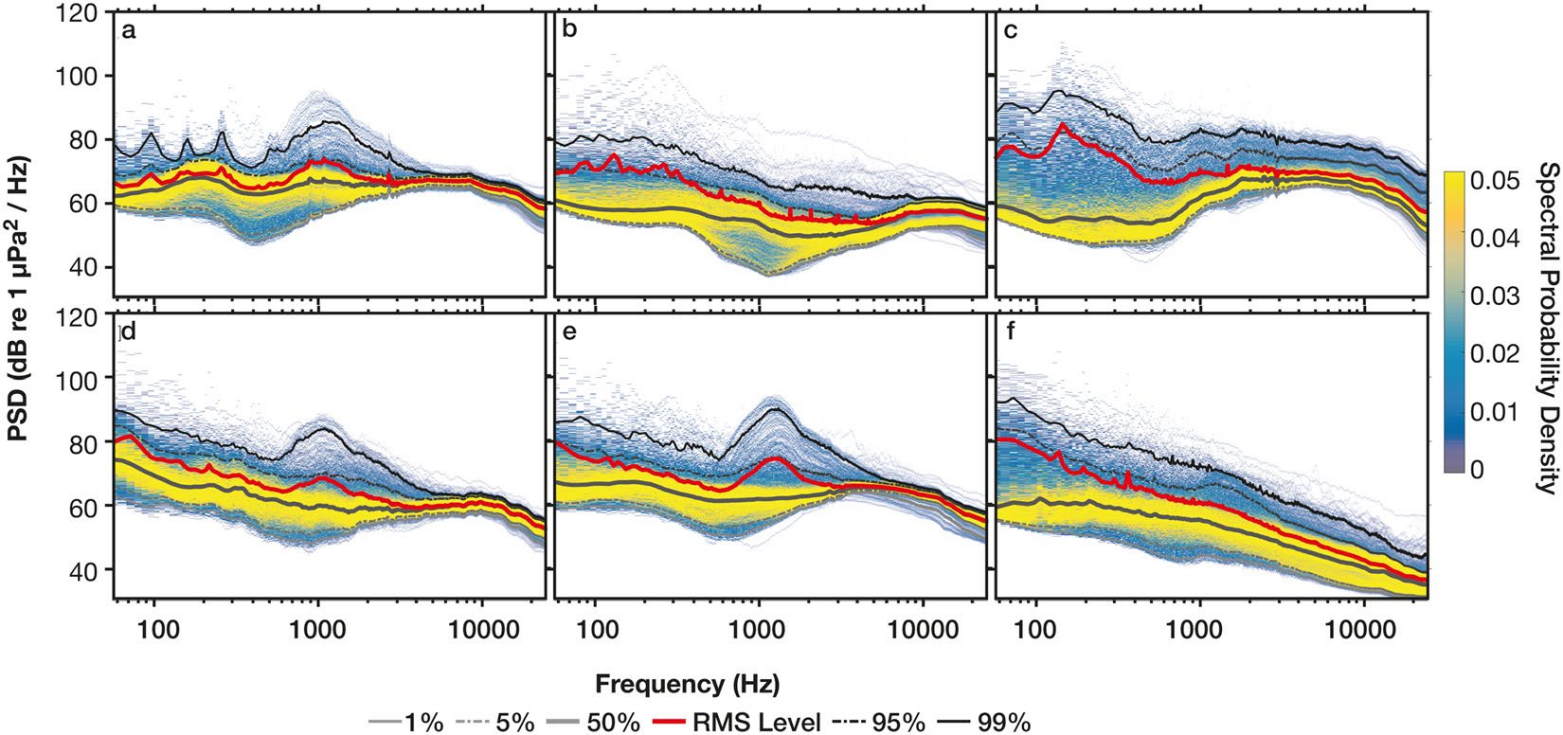
RMS level of the PSD, percentiles and spectral probability density (SPD)) for April 2015 for each of the six listening stations, (**a**) Horn Rock, (**b**) Waiheke Island, (**c**) Bean Rock, (**d**) Shearer Rock, (**e**) Flat Rock, and (**f**) Jellicoe Channel. Spectral probability density is shown by the colour-bar in each subplot.

Focusing on April 2015, median power spectral density (PSD) for all listening stations was very quiet ranging from 50–70 dB re 1 µPa^2^/Hz between 50 and 24,000 Hz frequency range (Fig. 2). However, the soundscape of each listening station consisted of different individual sounds, identifiable by reviewing percentile occurrence of different frequency ranges (Fig. 2). In the low frequencies (<200 Hz) the 99^th^ percentile PSD showed sound exceeded 90 dB re 1 µPa^2^/Hz at Bean Rock, 85 dB re 1 µPa^2^/Hz at Shearer Rock, Flat Rock and Jellicoe Channel and 80 dB re 1 µPa^2^/Hz at Horn Rock and Waiheke Island (Fig. 2) only 1% of the time. At all listening stations the spectral probability density (SPD) range was between 30 and 50 dB (below approximately 2 kHz) and three spectral peaks <400 Hz were noticeable when PSD levels were high (5^th^ percentile) at Horn Rock (Fig. 2a). Furthermore, daily spectrograms (Supplementary Fig. S2) showed high PSD levels (up to 90 dB re 1 µPa^2^/Hz) across the entire frequency range (up to 24,000 Hz) intermittently throughout the day but not during the night.

Another distinct feature in the soundscape during April 2015 was an increase in PSD across the 1–10 kHz frequency band at all listening stations except Jellicoe Channel, the deepest site (Fig. 2). At Bean Rock, for frequencies >3 kHz the difference between the 99^th^ and 1^st^ percentile was <10 dB (Fig. 2) suggesting a ubiquitous sound producing organism that is active equally throughout all times of the day and night (Supplementary Fig. S2).

During April 2015, at the rocky reef stations (Horn Rock, Shearer Rock and Flat Rock), the 1^st^ and 5^th^ percentile PSDs showed a distinctive increase on the order of 20–30 dB re 1 µPa^2^/Hz in the 600–3,000 Hz frequency band (Fig. 2a,d,e). At these listening stations, the maximum daily SPL occurred directly after sunset (lasting 1–3 hours) and a second peak occurred directly before sunrise (Supplementary Fig. S3). At Horn Rock, maximum SPL calculated in the 800–2,500 Hz frequency band in summer was 125.5 dB re 1 µPa compared to 113.1 dB re 1 µPa during spring. There was a significant positive correlation (Pearson’s Correlation r = 0.852, p < 0.001) between SPL and mean daily temperature at all stations (Supplementary Fig. 4), highlighting the seasonal trend in maximum SPL between 800–2,500 Hz.

### Geophony

**Figure 3:**
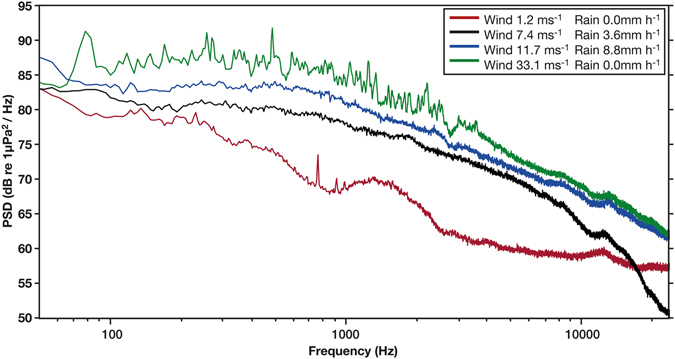
Power spectrum levels of four weather scenarios, produced using FFT length = 16384 points, Hanning window and 50% overlap. Red line - low wind speed 1.2 ms^−1^ and low rainfall 0.0 mmm h^−1^. Black line - medium wind speed 7.4 ms^−1^ and medium rainfall 3.6 mmm h^−1^. Blue line - high wind speed 11.7 ms^−1^ and high rainfall 8.8 mmm h^−1^. Green line - gale warning wind speed 33.1 ms^−1^ and low rainfall 0.0 mmm h^−1^. Each line represents a single event taken as an example of the corresponding weather scenario.

The different weather scenarios used showed that as wind and rain increased, PSD across the entire acoustic spectrum also increased (Fig. 3). During the deployment period when high rainfall was recorded there were also generally higher winds (67% of the time). Whereas, when there were high winds no rainfall was recorded (97% of the time). During a gale warning (wind 33.1 ms^−1^) when no rainfall occurred (Fig. 3) the PSD was higher across the entire acoustic spectrum compared to when both wind and rainfall was high; this was the case during all of the seven gale warnings issued during the deployment period.

Within the recording period there were 284 earthquakes >4 magnitude in New Zealand’s exclusive economic zone (EEZ). Thirty-two of these earthquakes occurred during the duty cycle of acoustic recordings and of these 12 were manually detected on at least one hydrophone. The other 20 earthquakes were not detected because vessel sound was present in each concurrent recording between 10–500 Hz, therefore the earthquake sound could not be distinguished and spectral characteristics could not be determined accurately. One 4.6 magnitude earthquake that occurred, on the 5^th^ June 2016, 790 kilometres away from Flat Rock had a peak frequency of 100 Hz, frequency bandwidth 890 Hz and sound duration of 4.2 seconds. Comparing broadband SPL (10–24,000 Hz), during the earthquake to that in the same recording immediately prior, earthquakes rose the ambient SPL at Flat Rock by 40 dB.

### Biophony

**Figure 4:**
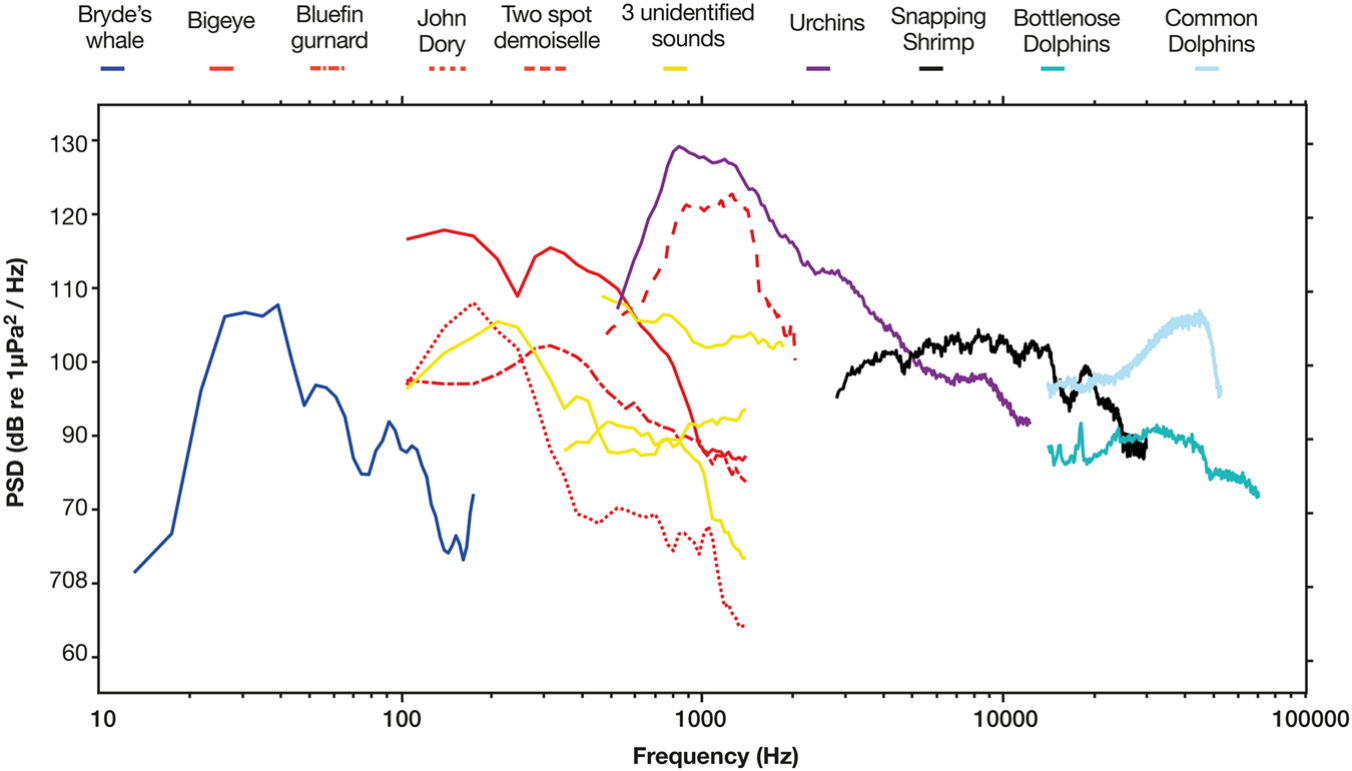
Power spectral density (dB re 1 µPa^2^Hz^−1^) for the best example of nine different biological sounds manually identified from recordings taken throughout the Hauraki Gulf.

Manual inspection identified nine different biological sounds that could be attributed to a distinct species. Each different taxon (whales, fish, urchins, snapping shrimp and dolphins) occupied a different frequency range (Fig. 4).

**Figure 5:**
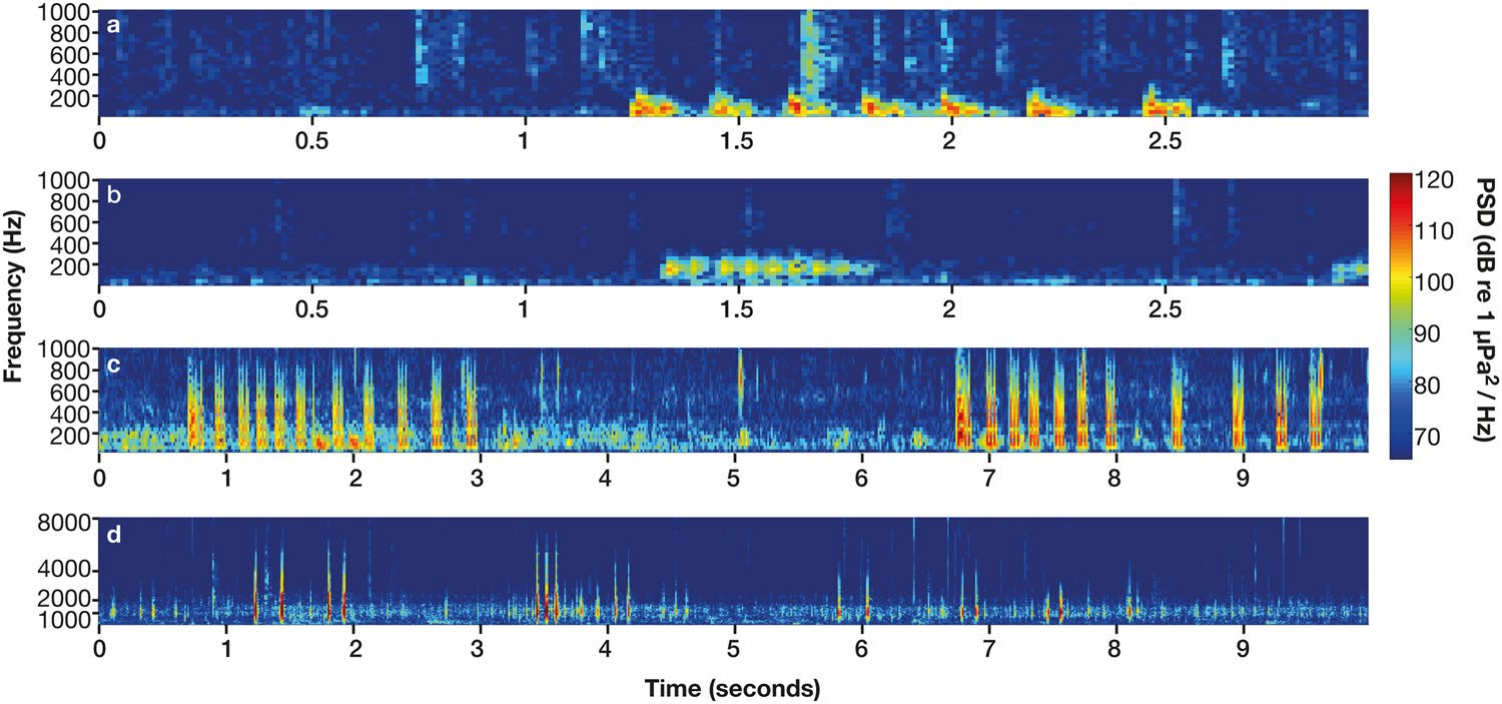
Spectrograms of four different fish species, (**a**) Bluefin gurnard “grunt”, (**b**) John Dory “bark”, (**c**) Bigeye “pops” and (**d**) two spot demoiselle “clicks”, all produced using FFT length = 1024 points, Hanning window and 50% overlap. The colour-bar shows the power spectral density (dB re 1 µPa^2^Hz^−1^). Note the different time and frequency scales.

We identified 858 individual vocalisations from four fish species; bluefin gurnard (*Chelidonichthys kumu*), John Dory (*Zeus faber*), bigeye (*Pempheris adspersa*) and two spot demoiselle (*Chromis dispilus*). Bluefin gurnard (Fig. 5a) produce a “grunt” consisting of short repeated pulses^29^. Those manually analysed in this study had a peak frequency of 106 ± 4 Hz and duration of 0.7 ± 0.04 seconds (n = 151). John Dory (Fig. 5b) produce a “bark” like sound in the 200–500 Hz frequency range^30^. In this study John Dory sounds had a peak frequency of 342 ± 6 Hz and duration of 0.4 ± 0.02 seconds (n = 298). Bigeye (Fig. 5c) emit “pop” like sounds in the 75–1000 Hz range^31^. In this study the sounds had a peak frequency of 220 ± 12 Hz individual pops had a duration of 0.9 ± 0.08 seconds (n = 114), individual bigeyes have been reported to repeat pops on average three times, and up to seven times, in succession^31^. Two spot demoiselle (Fig. 5d) emit “clicks” in the 1,000–2,000 Hz range (*personal observation*), with peak frequency at 1,100 ± 20 Hz and click trains (repetitions) were longer duration than bigeye at 1.4 ± 0.05 seconds (n = 252).

At Horn Rock, Shearer Rock and Flat Rock, bigeye, two spot demoiselle and urchin sounds were recorded during the same diurnal periods, dawn and dusk. Urchin sounds differed from bigeye sounds in their frequency band (800–2,500 Hz^7^ compared to 75–1,000 Hz) and average power (40 dB compared to 36.8 dB). Two spot demoiselle and urchin sounds overlapped in their frequency band (1,000–2,000 Hz); although they were distinguishable by the type of sound produced, two spot demoiselle sounds were clicks (increasing average power by 33 dB) whereas urchin sound was a continuous chorus^7^. Whereas, at Horn Rock, Waiheke Island and Flat Rock, the bluefin gurnard and John Dory signals occupied the same frequency band (100–200 Hz^29^ and 200–500 Hz^30^) as bigeye, however these sounds occurred at sporadic times throughout the day and night, not just dawn and dusk, and increased average power by 26.4 dB and 24.3 dB from ambient levels.

**Figure 6:**
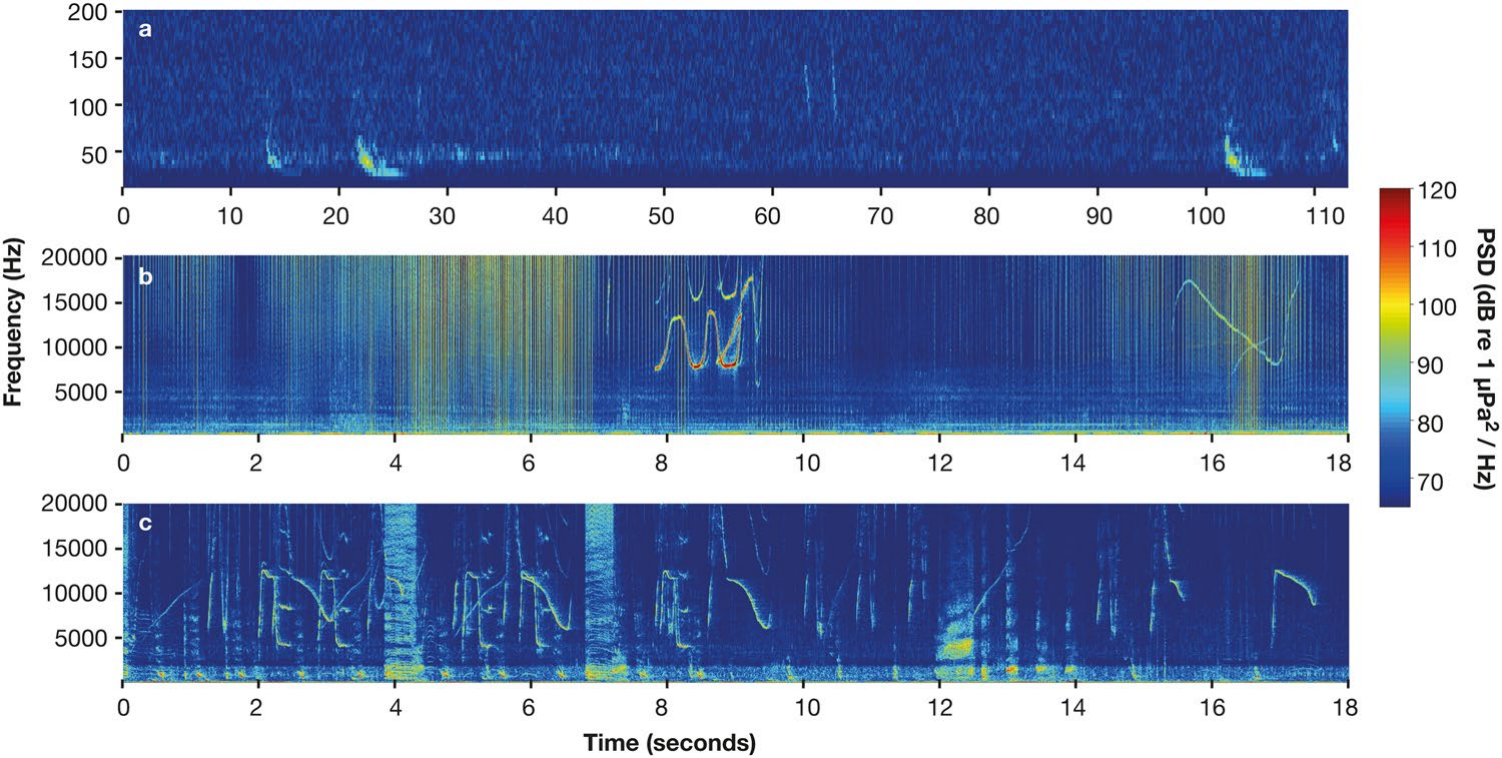
Spectrograms of three different cetacean species. (**a**) Bryde’s whale (*Balaenoptera edeni*) vocalisation downsampled to 8,000 Hz sample rate, FFT length = 1024 points, Hanning window and 50% overlap. (**b**) Common dolphin (*Delphinus delphis*) vocalisations FFT length = 512 points, Hanning window and 50% overlap. (**c**) Bottlenose dolphin (*Tursiops truncatus*) vocalisations FFT length = 512 points, Hanning window and 50% overlap. The colour-bar shows the power spectral density (dB re 1 µPa^2^/Hz).Note the different time and frequency scales.

At Jellicoe Channel and Flat Rock low frequency sounds were identified as Bryde’s whale (*Balenoptera edeni*) down-sweeps^32^. The down-sweeps ranged from 15–150 Hz (Fig. 6a), had a peak frequency of 35 ± 0.3 Hz (n = 45) and a duration of 2.6 ± 0.8 seconds. In addition to fish and whale sounds, eight other sounds were identified in the low frequencies (<3,000 Hz) during manual analysis from Horn Rock, a rocky reef listening station.

In the higher frequencies, snapping shrimp and dolphins dominated (2,000 Hz–10,000 Hz and, >10 kHz, respectively). Snapping shrimp “snaps” were recorded constantly at all sites except Jellicoe Channel. Dolphins were regularly recorded at the outer listening stations (Horn Rock, Flat Rock and Jellicoe Channel), but rarely at the inner sites (Waiheke Island, Bean Rock and Shearer Rock). Most frequently they were heard at Jellicoe Channel between 00:00 and 03:00. Two different species of dolphin: common (*Delphinus delphis*) and bottlenose (*Tursiops truncatus*) were identified in recordings (Fig. 6b,c) concurrent with surface observation on the days of hydrophone change over. During these recordings both species produced echolocation clicks >20 kHz, as well as a diverse range of whistles between 5–20 kHz (Fig. 6b,c).

### Anthropophony

**Figure 7:**
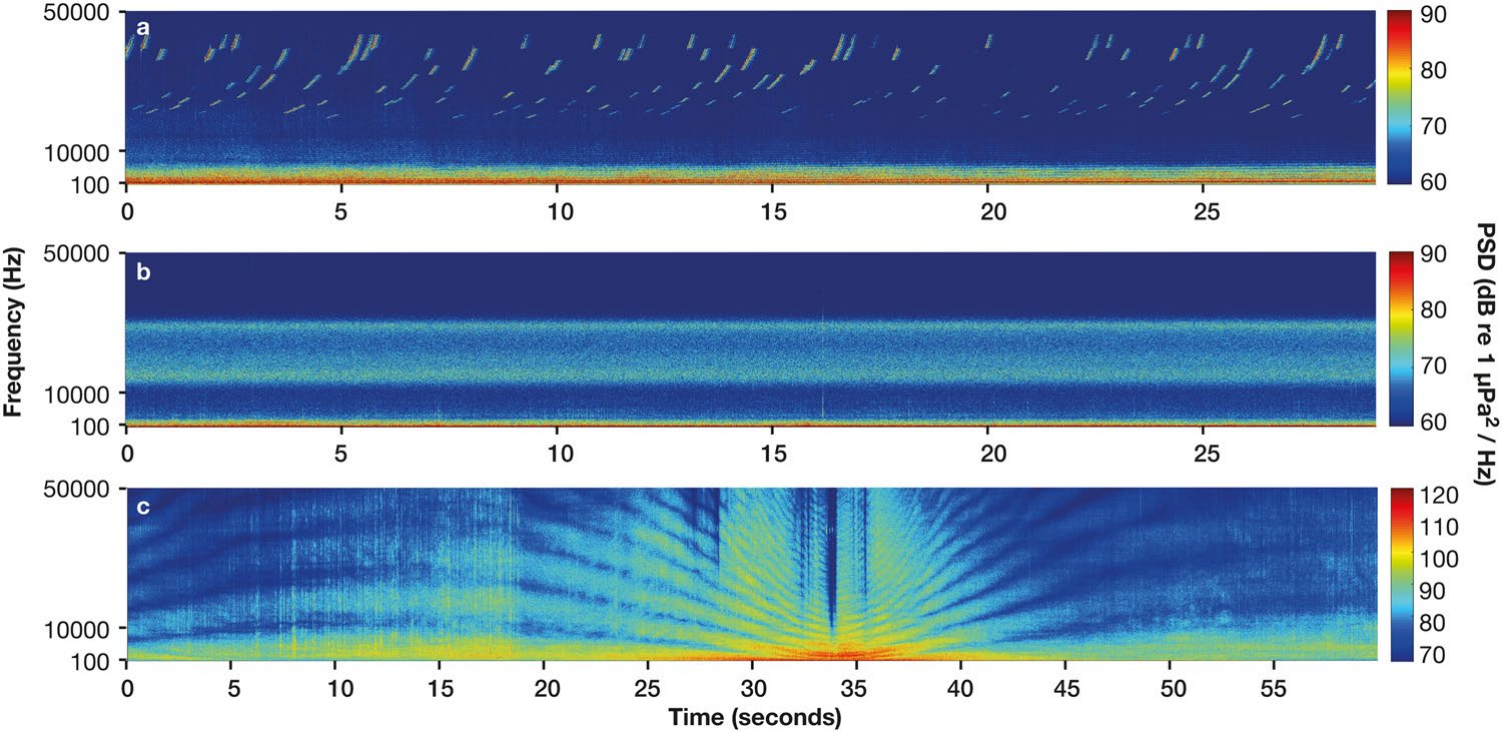
Spectrograms of three different anthropogenic sounds generated by shipping. (**a**) High frequency sonar produced by the echo-sounder, (**b**) High frequency cavitation produced by the propeller blade, (**c**) Low frequency engine sound. All produced using FFT length = 512 points, Hanning window and 50% overlap. The colour-bar shows the power spectral density (dB re 1 µPa^2^/Hz).

We identified three types of sound produced by vessel activity at all listening stations. Echo-sounder pings were within the frequency range 25–50 kHz, had a peak frequency of 30,560 ± 6,755 Hz, duration of 0.3 ± 0.1 seconds (n = 395) and increased ambient levels by 36 dB (Fig. 7a) whereas propeller cavitation was between 10–25 kHz, had a peak frequency of 20,485 ± 6,985 Hz (n = 120) and increased ambient levels by 30 dB (Fig. 7b). All propeller cavitation sounds lasted the duration of recording (two minutes), therefore total duration could not be evaluated. The most common anthropogenic sound detected was passing vessels, with the sound exhibiting the typical U-shaped Lloyd’s mirror pattern^33^ (Fig. 7c). Analysing all manually detected vessel passages, SPL increased by up to 60 dB between 50–10,000 Hz compared to SPL in the same recording immediately prior. Vessel passages were identified in 1.9–35.2% of the recordings manually inspected from Horn Rock (1.9%), Waiheke Island (11.5%), Bean Rock (35.2%), Shearer Rock (12.4%), Flat Rock (13.1%) and Jellicoe Channel (23.4%) (Supplementary Table S1). At Horn Rock (6.9%), Shearer Rock (18.8%) and Flat Rock (25.7%) there were slightly more vessel passages identified during spring and summer than the deployment averages (Supplementary Table S1). Additionally, listening station had a significant effect on vessel passage percentage (two-way ANOVA F_5_ = 50.465 p < 0.001) with Horn Rock having significantly less, compared to Jellicoe Channel and Bean Rock with significantly more vessel passages than the other three stations.

## Discussion

We investigated the use of recordings with high temporal resolution, a large spatial scale (tens of kilometres between listening stations) and high sampling frequency to understand the complex nature of soundscapes from a unique ecosystem. It allowed an improved appreciation of the complex interplay between factors that make up the marine soundscape. Previous low resolution (single sites and/or short duration) studies have shown that soundscapes vary temporally and spatially^2, 16, 20–22, 24, 25^. Following on from this research, a goal of the current study was to evaluate the relative importance of the contribution that both geophony and biophony has on the ambient soundscape. Different types of vessel sound raised the ambient sound level by up to 60 dB between 50–10,000 Hz, which overlaps the large majority of biophonic sources in this ecosystem’s soundscape potentially causing energetic masking^34^.

It is important to note, that depth can play a major role in what signals propagate in shallow water. Using normal mode propagation theory research showed the cutoff mode in 50 m of water was 22 Hz for the Hauraki Gulf^35, 36^. At Jellicoe Channel, positioned at 57 m water depth, the Bryde’s whale vocalisation was the lowest frequency sound recorded (peak frequency 35 Hz) and would therefore not be affected. Whereas, in 10 m of water the cutoff for mode 1 was approximately 110 Hz, therefore at Bean Rock, positioned at 6 m water depth, low frequency sounds <150 Hz may be distorted which could affect whale vocalisations and vessel passage sound (50–10,000 Hz). The other four listening stations (Waiheke Island, Horn Rock, Shearer Rock and Flat Rock) were positioned on or near rocky reefs (18–20 m), ergo biophony was more diverse with marine mammals, fish and invertebrates recorded, and only frequencies below 60–80 Hz might have been affected by normal mode cutoff frequency.

In order to compare between soundscapes at different locations propagation conditions need to be considered, especially water depth. For example, the PSD (10–100 Hz) for the Perth Canyon (>400 m water depth) was found to be between approximately 75–100 dB re 1 µPa^2^/Hz, whereas the PSD (50–200 Hz) recorded in this study at Horn Rock (18 m water depth) was lower ranging between 60–80 dB re 1 µPa^2^/Hz (Fig. 2a). Due to the big difference in water depth between the two studies the cutoff frequencies are also going to be significantly different making comparison difficult. Furthermore, it had previously been suggested that shallow embayments with a relatively constant depth seafloor, such as the Hauraki Gulf, would allow for much better propagation of distant shipping noise compared to areas of irregular bathymetry^37^. However, the PSD levels recorded at Horn Rock (50–200 Hz) were similar to the PSD levels (10–200 Hz) recorded around San Clemente island, southern California, 55–95 dB re 1 µPa^2^/Hz, despite the area having irregular bathymetry including deep canyons (<110 m water depth)^37^. The low sound levels recorded in Hauraki Gulf were also comparable to other shallow water locations worldwide^37–39^. Sites in the Arafura and Timor Sea had low ambient noise levels of approximately 50–80 dB re 1 µPa (50–10,000 Hz)^38^, and the RMS SPLs (50–24,000 Hz) recorded at all listening stations and those in the Stellwagen Bank National Marine Sanctuary^39^ (10–1,000 Hz), were both less than 100 dB re 1 µPa (Fig. 1). Even including the presence of local shipping sound in 1.9–35.2% of recordings (Supplementary Table S2), at each listening station geophony and biophony components were deciphered from each individual soundscape.

New Zealand is renowned for being tectonically active because of the country’s location on the boundary of the Australian and Pacific tectonic plates. Earthquakes were detected up to 800 kilometres away from their source and raised the ambient soundscape by up to 40 dB however, it is unknown if the sound of earthquakes affects marine animals. At all listening stations the diverse range of biophony and anthropophony have the potential to be able to mask earthquakes. Importantly, the sound was also a rare feature in the long-term soundscape with only 12 earthquakes (>4 magnitude) detected between all listening stations’ recordings, therefore perhaps here and in other similar biologically diverse shallow embayments, geophony from earthquakes may not be a key contributor. Whereas, the weather was considered to be a relevant contributor to the geophony because as wind-speed and rainfall increased the PSD also increased (Fig. 4). The weather is important in shallow environments because the sound from the surface is able to propagate to the seafloor^40^, resulting in the possibility that PAM could be a cost effective and efficient way of monitoring offshore weather^28^.

Different habitats produce unique soundscapes due to the organisms living within the unique acoustic properties of the environment^20–22^, and therefore each listening station had an individual soundscape. At Jellicoe Channel, the deepest listening station, sporadic fish, dolphin and Bryde’s whale vocalisations dominated the soundscape. Because the habitat was deep sand (with a high impedance ratio that refracts sound), low frequency biological sounds (e.g. Bryde’s whales) may have been detected from afar^41^. Whereas, the shallowest site, Bean Rock has a low impedance ratio because the deep mud absorbs sound^41, 42^, hence any sounds recorded were produced close by. At Bean Rock, the RMS SPL was significantly higher during the day because of near constant sound from passing vessels. At Bean Rock there was also clear rise in PSD between 2–10 kHz (Fig. 1c), indicative of the ubiquitous snapping shrimp^10^ which was also a dominant feature of the soundscape throughout the entire recording period. In comparison, despite having been documented in waters less than 60 m^10^, snapping shrimp were not recorded at Jellicoe Channel.

At the rocky reef listening stations, RMS SPL varied according to season and time of day, with the RMS SPL significantly higher during dusk and summer than other respective times of day or season. The dominant feature of biophony at the rocky reef listening stations was the routine rise of PSD levels in the 800–3,000 Hz bandwidth during dawn and dusk periods (Fig. 1, Supplementary Figs 3 and 4), consistent with previous studies in both temperate and tropical waters^15, 20^. In New Zealand, the source has previously been identified as a sea urchin chorus^43^. In this study the crepuscular chorus was recorded during the austral spring and summer but to a lesser extent in autumn and winter, suggesting urchins, are either no longer present and/or producing sound during autumn and winter. Urchins have large seasonal fluctuations in algal consumption with higher feeding rates noted in the summer^44^. High feeding rates reflect a higher energy requirement for reproduction and growth, and can indicate the food quality and availability at a particular site^45^. Kelp biomass and photosynthetic rates are reduced during the winter months on temperate reefs in New Zealand^46^, indicating less food is available for grazers like urchins and herbivorous fish. Notably, the sound level increase precedes the temperature increase, however the gradual seasonal change in sound level (Supplementary Fig. S4) indicates that some urchins still feed (known because the sound is produced by the feeding mechanism^7^) whilst others cease, because there is still food available (there is not a complete eradication of kelp in winter) and that there may be intraspecific variation in thermal tolerance among populations, individuals or life stages^47^. In future, the sound of a biological chorus could potentially be used as a proxy of feeding activity for urchins at rocky reefs. Importantly, rising water temperatures may also cause feeding activity to occur year-round, and hence the seasonal variation in sound pressure levels to cease.

Unlike urchins and snapping shrimp, the reason for different fish vocalisations remains unclear. Some fish were heard sporadically throughout the day and year whereas others were heard at the same time every day. John Dory and bluefin gurnard vocalisations were irregular which could be linked to their behavioural function^29^. Bigeyes and two spot demoiselles were recorded at dawn and dusk. Bigeye vocalisations were recently linked to group cohesion in the schooling fish^13^ and this could also be the case for the two spot demoiselle. Four different types of fish vocalisation were positively identified to their species yet, over 80 different fish species reside in the Hauraki Gulf and the eight unknown vocalisations identified in this study could belong to some of them. Fish choruses off Port Headland, Australia have also exhibited various combinations of temporal and frequency partitioning^48^. Identifying key acoustic traits of different species of fish is important because it allows improved understanding into their patterns of recruitment, migration and habitat use.

The sounds of whales and dolphins were a major contributor to the overall soundscape. The three different marine mammal species recorded in this study are resident to the Hauraki Gulf year round, although there is limited literature about their acoustics within the embayment^32, 49^. Bryde’s whales were the only biological source below 100 Hz, and the dolphin echolocation clicks were the only biological source exceeding 20 kHz (Fig. 6). Detected vocalisations provide an insight into visitation patterns of cetaceans in the Hauraki Gulf. Whales and dolphins were heard throughout the day at the outer listening stations (Horn Rock, Flat Rock, and Jellicoe Channel) and dolphins were regularly heard between midnight and dawn at Jellicoe Channel. At the inner listening stations (Bean Rock, Shearer Rock and Waiheke Island) dolphin recordings were rare and Bryde’s whales were never recorded. The presence of whales however cannot be eliminated because first, they do not continuously produce sounds so their vocalisations may have been missed by the recorders’ duty cycle and second, anthropogenic sound is prevalent in low frequencies, therefore it is unknown if Bryde’s whale sounds were masked.

Different taxon (whales, fish, urchins, snapping shrimp, dolphins) in the present study consistently held their own aural niche within the soundscape (Fig. 4) supporting the acoustic niche hypothesis (ANH) that different sources have their own signature frequency. There is overlap in biophony between 100 to 1,000 Hz, because of multiple fish species vocalisations. Different fish vocalisations also fit the acoustic niche hypothesis because they are heard at different times^50^ or occupy different frequencies when heard at the same time. Because the different elements of biophony are known, long-term passive acoustic monitoring has the potential to provide a cost effective and autonomous way of monitoring animal populations, specifically their visitation patterns and relative abundance^28^. Importantly, ANH posits that the sound spectrum is a limited resource and that species try to minimise acoustic competition^51^. Each major contributor, Bryde’s whales, fish, urchins, snapping shrimp and dolphins were all clearly partitioned in the frequency spectrum (Fig. 4). The ANH indicates a healthy ecosystem would be clearly partitioned into niches by frequency or time. In contrast, a disrupted area would have gaps, where acoustic signals of species have been lost or masked by other sounds^50^. Differences in the timing and frequency of different sounds if linked to specific characteristics, such as fish abundance, could thereby indicate the health or productivity of an area.

A major concern facing scientists and policy makers alike is sound pollution from vessels because the sound they produce provides a major contribution to the overall soundscape^52^. The Hauraki Gulf provides an excellent case study because in this ecologically significant embayment anthropogenic activity is increasing. Since 1994, visits of vessels >9000 GWT to Auckland have grown by over 23% (BECA, 2012) and the number of recreational vessel owners in the Hauraki Gulf is predicted to rise by 25% in the next twenty years (BECA, 2012). Vessel sound not only contributed to elevated levels at low frequencies (<200 Hz) (Fig. 1) across all listening stations but intermittently across the entire frequency range (50–24,000 Hz) throughout the day and night. A key finding was when a vessel was present the ambient sound level rose by up to 60 dB between 50–10,000 Hz and 30 dB between 10,000–24,000 Hz (Fig. 7). Currently the potential impacts of increasing anthropophony are relatively unknown. Many species of fish and invertebrates have evolved to use geophony and biophony as an orientation cue for selecting suitable habitats during larval recruitment^53^. While other marine animals make use of sound for everyday life, for communication, finding food and potential mates. Tagging showed that Bryde’s whales in the Hauraki Gulf vocalise infrequently but are frequently exposed to high levels of continuous background ship noise^32^. The reasons for low vocalisation rates are currently unknown but the potential for masking is high given the overlap in frequency ranges between the whale vocalisations and ships. Future research is warranted to determine the spatial extent of vessel sound in the Hauraki Gulf as well as its capability to alter or indeed mask biophony.

Following the ANH, soniferous species would adjust their signals in order to minimise interference^50^ from anthropophony. However, realistically, there are limited options available to compensate for increased sound in the ocean. Some animals increase the volume of their vocalisations^54, 55^, to be heard over noise, exhibiting the Lombard effect. Other animals alter their acoustic characteristics, by shifting signal frequencies, making them longer^56^ or waiting until anthropogenic sound ceases before vocalising^57^. In the Hauraki Gulf soundscape niches in frequency band were found between taxa and differences in the timing and structure of sounds were found within taxa, so there may be limited space for sound sources to shift in the frequency or time domains. Acoustic and behavioural changes in cetaceans have been linked to chronic stress^58^, as well as potential repercussions in reproduction success and population survival^59^. However, the consequences of increased anthropogenic sound and potential masking of biophony produced by fish and invertebrate species is largely unknown.

Knowledge of soundscape ecology can provide managers tools to address concerns about the rising levels of anthropogenic sound. Other ecologically significant embayments for which vessel activity is also a common feature include Stellwagen Bank in the USA^39^ and Haro Strait, Canada. Vessel sound has been modelled to inform marine spatial planning including potential impacts on aquatic life^39, 60^. Both areas are home to a diverse range of soniferous fish and cetaceans, however are subject to heavy vessel traffic into the ports of Boston^39^, Vancouver and Seattle^60^. The Hauraki Gulf is still a relatively quiet embayment when compared to sound analysis from these areas, therefore this study provides a baseline against which future vessel activity in the embayment could be assessed.

## Conclusion

In conclusion, the Hauraki Gulf soundscape is complex and composed of rare geophonic and regular biophonic sources. The various sources of biophony have been found to hold their own acoustic niche either in the frequency or time domains. Sources of geophony and anthropophony, whether it be heavy rain, strong winds, or commercial shipping, all have the ability to raise the sound level of the ambient soundscape. Anthropophony continues to increase in the world’s oceans and future research directions will need to address concerns about whether anthropophony may mask biophony, which is crucial for life history strategies of many marine animals. Understanding the current soundscape in ecologically significant embayments, like the Hauraki Gulf, around the world is the first step in determining any potential consequences.

## Materials and Methods

### Acoustic data

**Figure 8:**
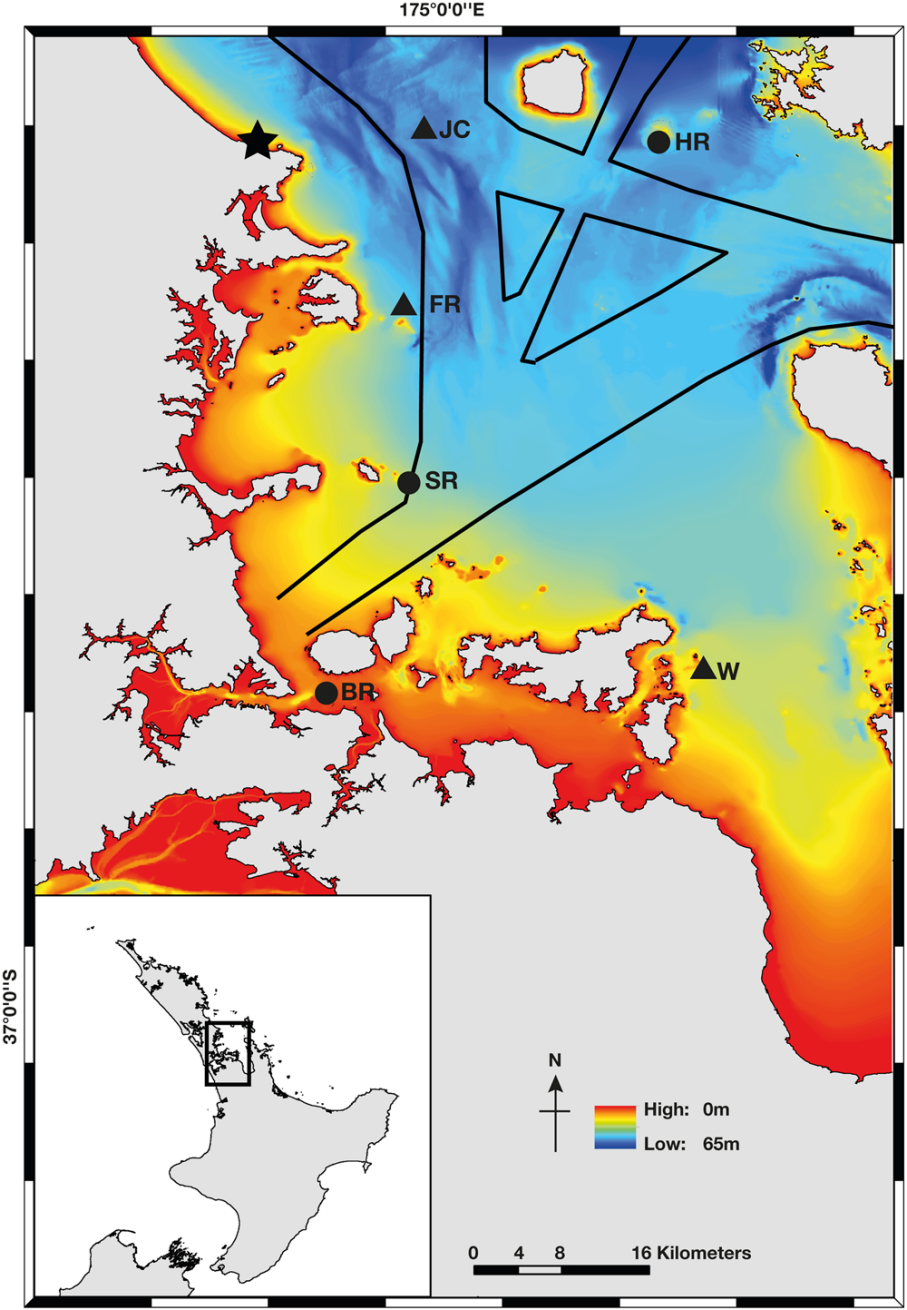
Map of the Hauraki Gulf, New Zealand showing the six listening stations: (clockwise from the top right) Horn Rock (HR), Waiheke Island (WI), Bean Rock (BR), Shearer Rock (SR), Flat Rock (FR) and Jellicoe Channel (JC). Circles represent diver retrieval and triangles acoustic release retrieval. The star represents the location of the Leigh Marine Laboratory weather station. Bathymetry of the area is also shown by the colour-bar from 0–65 metres and the black lines depict an outline of routes taken by vessels >70 m in length between July 2012 and June 2013^32^. Map produced using ArcGIS 10.3.1 (http://www.esri.com/software/arcgis/).

The study was conducted in the Hauraki Gulf, New Zealand; a large, island studded embayment recognised for its high biodiversity value (over 700 marine intertidal invertebrates, 80 species of fish and 4 cetacean species are reliably found in the area^61^). It is also regularly used by both recreational and commercial vessels^62^. Acoustic data were collected at six listening stations around the Hauraki Gulf (Fig. 8, Supplementary Table S2), from October 2014 to June 2016 using omnidirectional acoustic recorders (ST202 Ocean Instruments, NZ). Deployment locations were chosen to encompass different temperate coastal habitats, water depth (the Hauraki Gulf has a maximum depth of 60 m^61^) and proximity to the most traversed shipping routes into the Ports of Auckland^32^. At three listening stations: Shearer Rock, Bean Rock and Horn Rock, recording apparatus (containing hydrophone, battery, recorder and timer) was attached to a weighted stand one metre off the seafloor, and retrieved by a diver. At the other three stations: Flat Rock, Jellicoe Channel and Waiheke Island, the recording apparatus was suspended two metres off the seafloor and retrieved using an acoustic release (Desert Star Systems). At each station an acoustic recorder was deployed ten times, with the exception of Flat Rock with eleven and Waiheke Island with five deployments (owing to loss of equipment). Each deployment lasted approximately 54 days.

At all listening stations, the acoustic recorder was programmed to sample at 144 kHz, 24 bit, for two minutes every twenty minutes for the duration of each deployment. All acoustic recorders were calibrated prior and post deployment using a piston phone.

### Soundscape analysis

All acoustic data were analysed using MATLAB software (version 2014a). The data were high pass filtered at 50 Hz to remove any potential surface motion noise or low frequency electrical interference.

To determine whether sound level varied over the duration of recordings four quantitative measures were calculated for each filtered recording: broadband (50–24,000 Hz) median, 95^th^ and 5^th^ percentile sound pressure level (SPL) as well as root-mean-square (RMS) SPL. Analysis up to the Nyquist frequency 72,000 Hz provided no additional biophony, geophony or anthropophony, therefore 24,000 Hz was deemed a suitable cut-off high frequency.

Each recording was assigned a time category (dawn, day, dusk or night). Dawn and dusk were recordings ±90 minutes sunrise and sunset respectively. The sunrise and sunset times, and lunar phase cycle data were obtained from the Royal Astronomical Society of New Zealand (http://rasnz.org.nz/in-the-sky/sun-rise-and-set). Quantitative measures were then averaged according to their assigned time category for each day, to show diurnal variation.

To determine if season, time of day or listening station effected the ambient sound recorded, RMS SPL at each listening station was analysed statistically using a three way ANOVA to assess the effect of each factor as well as interactions. A general linear model (GLM) was then applied fitting the data to a Poisson distribution. Then a predicted means analysis was performed the data outputted from the GLM to determine differences between RMS SPL for each interaction according to listening station, season and time of day.

To determine whether sound level varied with frequency, RMS SPL and power spectral density (PSD) was calculated for all filtered recordings between 50 and 24,000 Hz. PSDs were calculated using a fast Fourier transformation of 30 second samples, creating 1 Hz frequency resolution and applying a Hanning window with a 50% overlap. To evaluate data quality, monthly spectral probability density (SPD)^63^ (between 50–24,000 Hz) was calculated using all the recordings available for each month during deployment. A more comprehensive analysis of the sound level distribution is given by the spectral probability density. The probability density of sound levels in each frequency band is presented and shows the modal structure and outlying data in the underlying distribution, helpful when interpreting averages and percentiles^63^. April 2015 data is presented as all listening stations were in operation for the entire month.

Spatial and temporal patterns were reviewed by comparing the RMS and PSD levels across the listening stations at various time scales (day, week, month, season and annual). Spectrograms were produced for three days over each new moon during deployment. A period of three days was chosen to depict the standard daily patterns for each listening station, and the new moon was chosen as it has been recognised as the most intense period in the lunar calendar for temperate reef ecosystems^15^. The ecological habitat surrounding each listening station (Supplementary Table S2) was defined according to information in the Hauraki Gulf Marine Spatial Plan.

In addition to broadband analysis, maximum sound pressure level between 800–2,500 Hz was also calculated for every recording to assess for seasonal trends in sea urchin biological choruses on the rocky reefs^15^. Water temperature was logged every 20 minutes ( ± 0.1 °C) (Hobo Water Temp Pro v2) at the same depth and location as each acoustic recorder. A Pearson’s correlation coefficient was then used to assess the correlation between water temperature and the calculated maximum sound pressure level between 800–2,500 Hz.

### Weather scenarios

To assess how environmental variables alter the soundscape of the Hauraki Gulf weather data (wind speed, wind direction, rainfall rate) was collected from the weather station located at the Leigh Marine Laboratory for the duration of deployment. The data was received as hourly averages and because of the distribution of the data, wind rate was classified as low (<5 ms^−1^), medium (5–10 ms^−1^) or high (>10 ms^−1^) and rainfall as low (<2 mm hour^−1^), medium (2–4 mm hour^−1^) or high (>4 mm hour^−1^). PSD of concurrent sound, at the closest listening station (Jellicoe Channel), was used to visualise the acoustic spectrum of rain and wind for different weather scenarios between 50 and 25,000 Hz. Examples of low wind-speed/low rainfall, medium wind-speed/medium rainfall, high wind-speed/high rainfall and high wind-speed/low rainfall were used to investigate how increasing wind-speed and rainfall impacted the recorded sound level. The scenarios represented 67%, 2%, 1% and 19% of total deployment hours respectively.

### Manual Inspection of Acoustic Data

To determine the different types of geophony, biophony and anthropophony that occur in the Hauraki Gulf soundscape recordings from every full and new moon during deployment (176 days) were inspected both aurally and visually using scrolling spectrograms (first using FFT length = 1024 and then using FFT length = 16384). Manually identified sounds (geophony, biophony and anthropophony) were then analysed using RavenPro (version 1.5) to determine the minimum, 5^th^ percentile, 95^th^ percentile, maximum and peak frequency (Hz), as well as start, end and 90^th^ percentile duration (seconds) and average power (dB). Average power (dB) was also calculated for the remaining amount of time in the recording prior and post the detected sounds. Average PSD (dB re 1 µPa^2^/Hz) for each source of biophony was calculated and used to compare frequency partitioning in relation to the acoustic niche hypothesis.

In addition to full and new moon analysis, all concurrent acoustic recordings to earthquakes with a magnitude greater than four within New Zealand’s exclusive economic zone (in total 32 recordings) were also manually analysed using RavenPro to measure the same characteristics. Earthquake data were retrieved from the GeoNet Project (http://quakesearch.geonet.org.nz/). Due to the high frequency of earthquakes around New Zealand, only earthquakes with a magnitude >4 were used in the analysis.

To determine whether there was any seasonal difference in the acoustic presence of vessel passage sound, the percentage of recordings manually inspected that contained vessel sound for each listening station and season were analysed using a two way ANOVA.

## Electronic supplementary material

Supplementary Information
