# Supplementary material for: Exploring spatial and temporal trends in the soundscape of an ecologically significant embayment
Source: Sci Rep. 2017 Jul 18;7:5713. doi: 10.1038/s41598-017-06347-0 (PMC5516011; doi:10.1038/s41598-017-06347-0)
Supplement: Supplementary file 1 — Supplementary Information [file 41598_2017_6347_MOESM1_ESM.pdf]

Exploring spatial and temporal trends in the soundscape of an ecologically significant embayment

Putland, R.L[a*], Constantine, R.[b], Radford, C.A.[a]

[a] Leigh Marine Laboratory, Institute of Marine Science, University of Auckland, PO Box 349, Warkworth, 0941, New Zealand

[b] School of Biological Sciences, University of Auckland, Private Bag 92019, Auckland, 1142, New Zealand

[*] Corresponding Author: rput037@aucklanduni.ac.nz

+64 9 923 3620

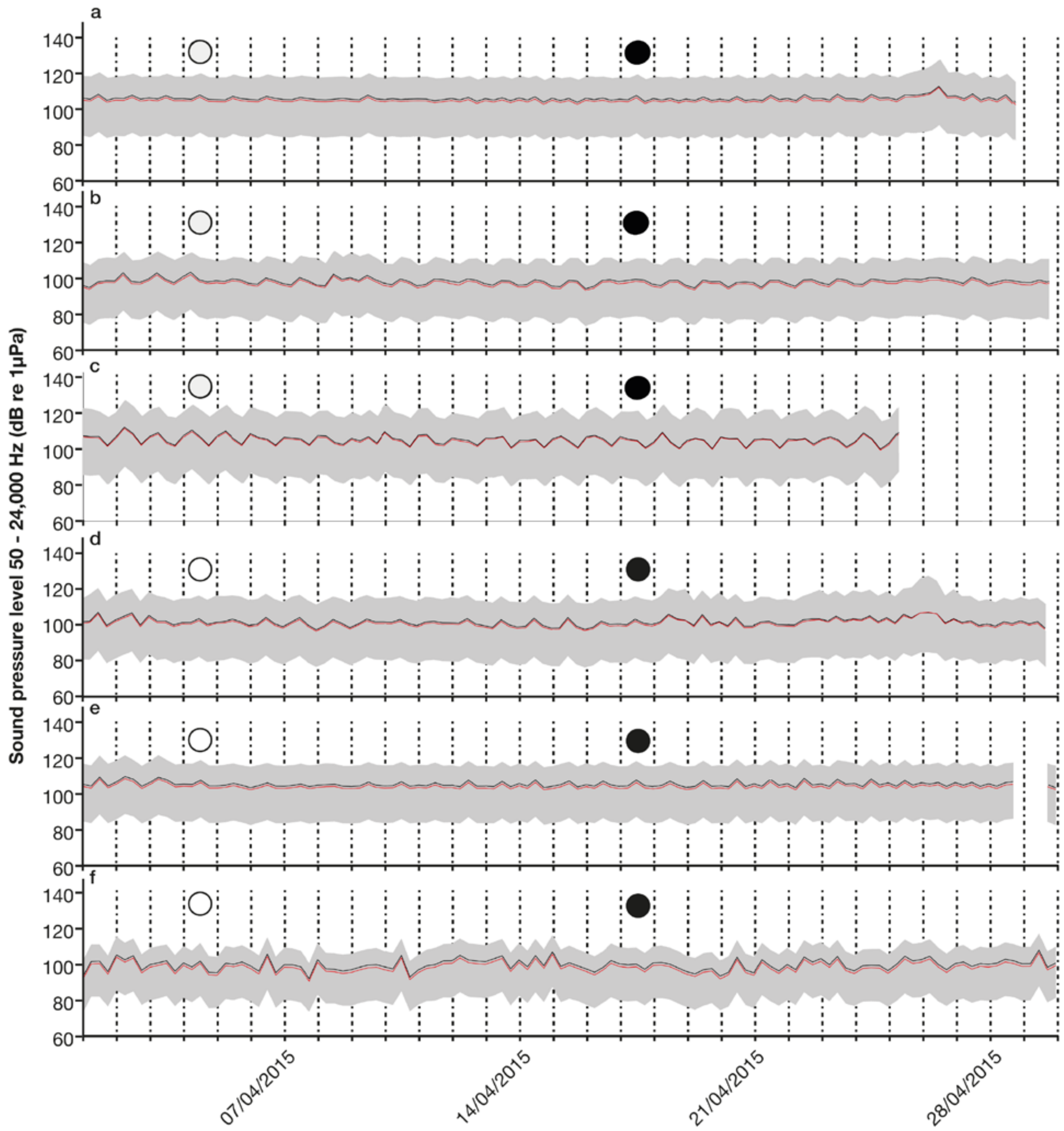

Supplementary Figure S1: RMS level (red line), median (black line), 5th and 95th percentiles (shaded area) of broadband sound pressure level between 50-24000Hz for daily (day, dawn, dusk and night) time categories over April 2015 for each of the six listening stations, a) Horn Rock, b) Waiheke Island, c) Bean Rock, d) Shearer Rock, e) Flat Rock, and f) Jellicoe Channel.

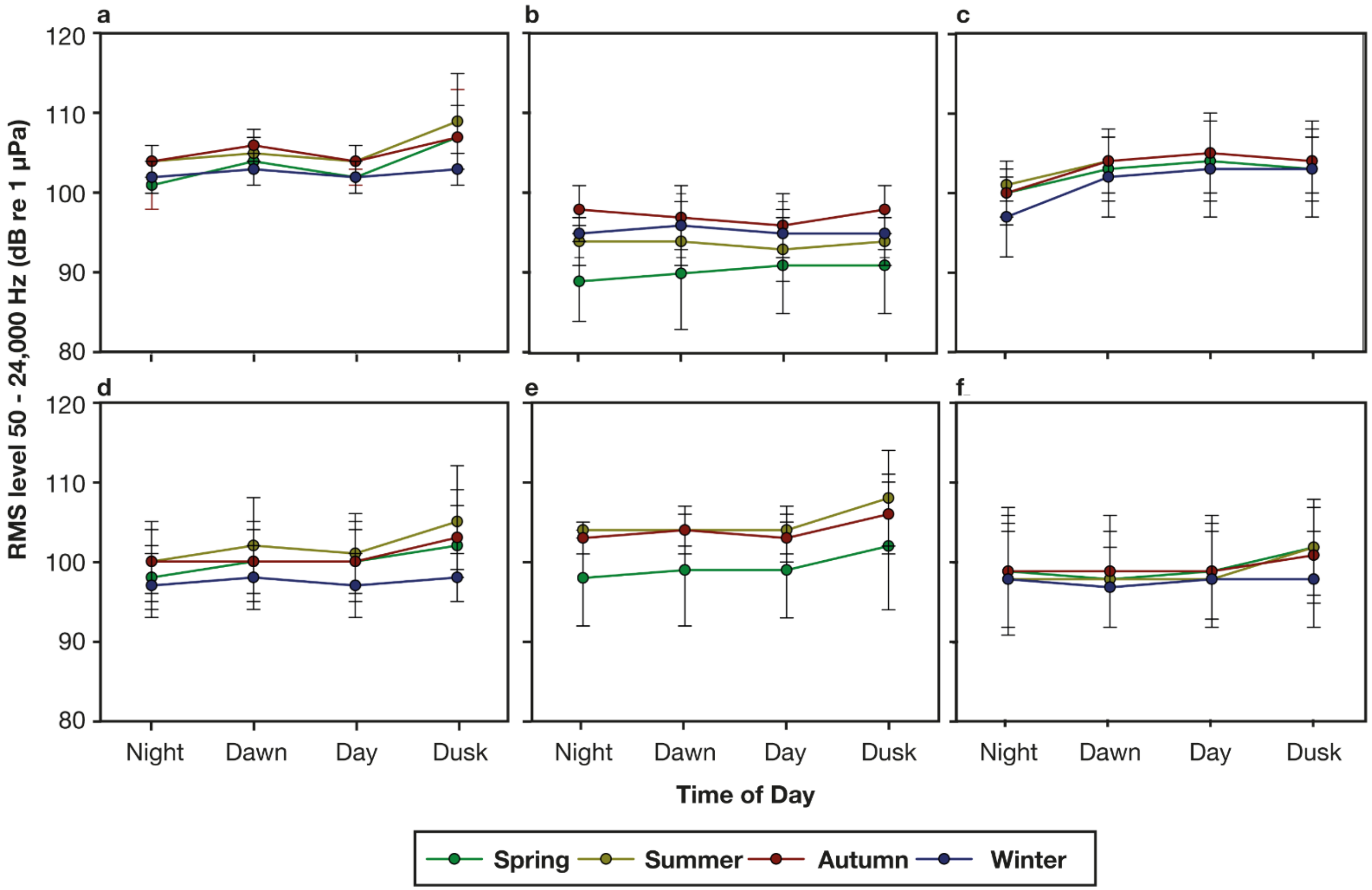

Supplementary Figure S2: RMS level 50 – 24,000 Hz (dB re 1μPa) of all recordings throughout period according to season and time of day for each of the six listening stations: a) Horn Rock, b) Waiheke Island, c) Bean Rock, d) Shearer Rock, e) Flat Rock and f) Jellicoe Channel.

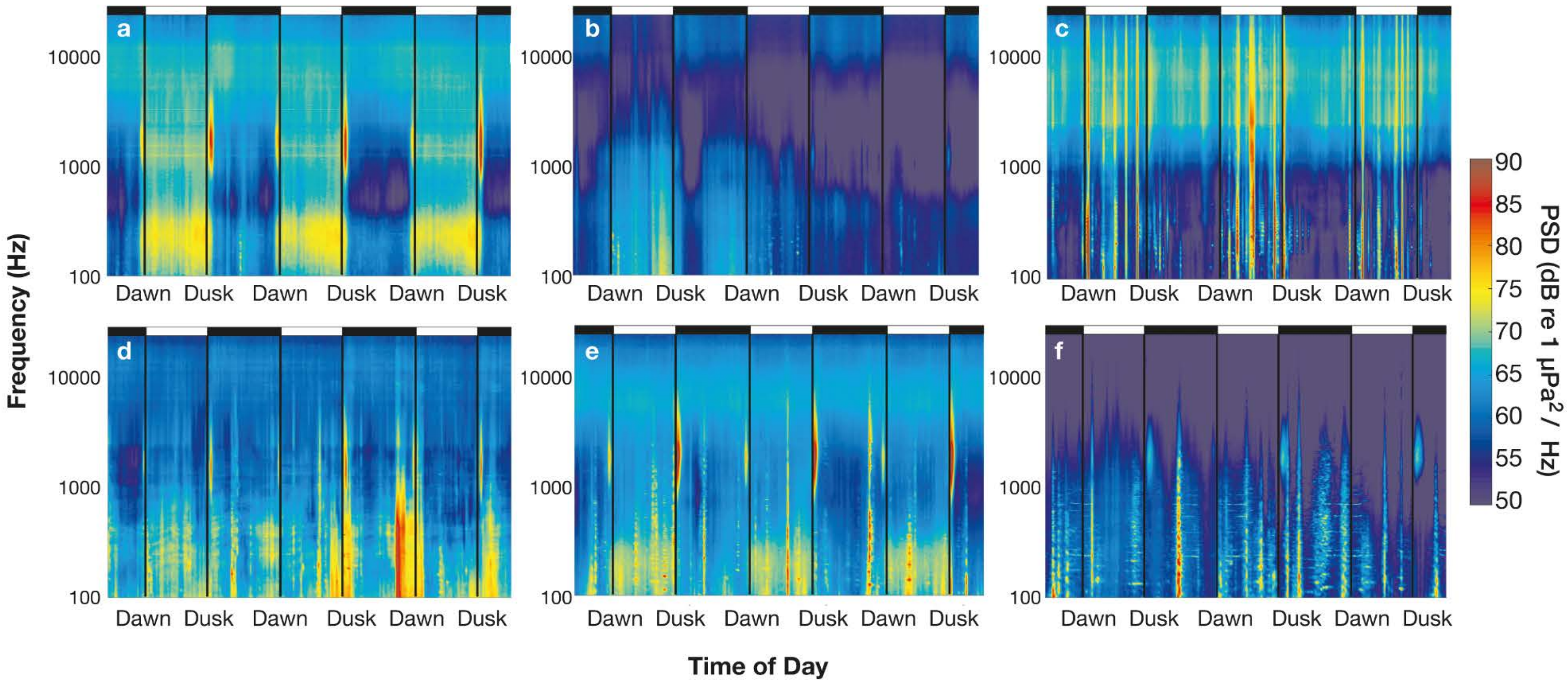

Supplementary Figure S3: Spectrograms of the three days around new moon from each of the six listening stations: A) Horn Rock, B) Waiheke Island, C) Bean Rock, D) Shearer Rock, E) Flat Rock, and F) Jellicoe Channel. Black bars at the top of each figure represent night. Power spectral density (dB re 1μPa Hz^{-1}) in each subplot is shown by the colour-bar.

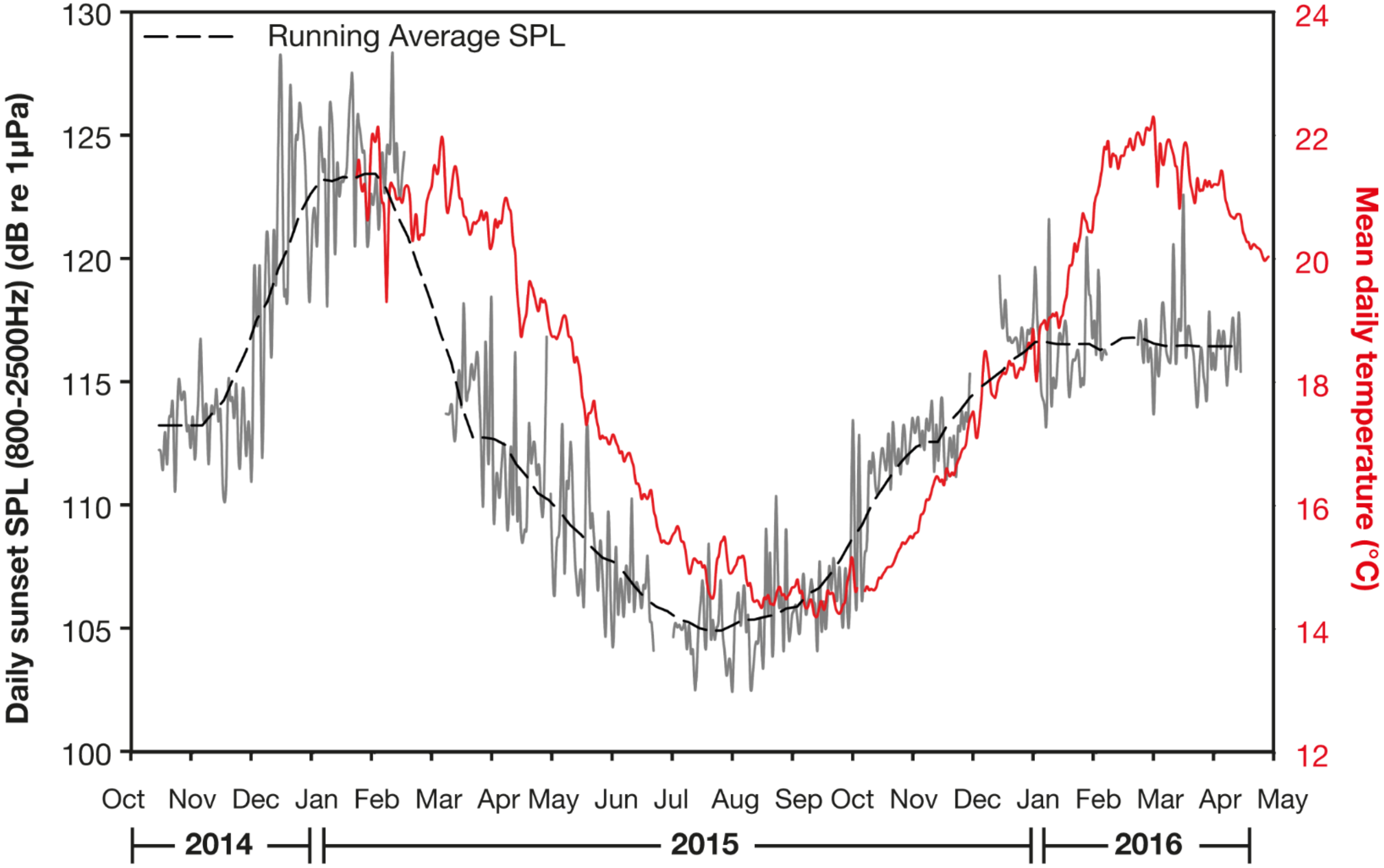

Supplementary Figure S4: Mean sound pressure level (dB re 1µPa) between 800 and 2500 Hz taken at sunset each day from the Horn Rock recording from October 2014 to April 2016. The black dotted line represents the running average SPL calculated using a 3 sample day window. Secondary plot (red) is of mean daily temperature (^{0}C) also at Horn Rock for the same time frame.

Supplementary Table S1: Percentage (%) of two-minute recordings manually inspected that contained vessel passsage sound (number of recordings manually inspected in brackets).

	**Horn Rock**	**Waiheke Island**	**Bean Rock**	**Shearer Rock**	**Flat Rock**	**Jellicoe Channel**
**2014**	**2.8 (n= 360)**	**12.85 (n= 288)**	**34.4 (n= 360)**	**17.8 (n= 360)**	**17.5 (n= 288)**	**30.3 (n= 432)**
Spring	3.7 (n= 216)	13.9 (n= 216)	35.7 (n= 216)	17.1 (n=216)	12.0 (n= 144)	25.9 (n= 216)
Summer	1.4 (n= 144)	9.7 (n= 144)	32.6 (n= 144)	18.8 (n= 144)	25.7 (n= 144)	36.8 (n= 144)
**2015**	**2.6 (n= 1224)**	**9.5 (n= 504)**	**33.3 (n= 1584)**	**10.4 (n= 1512)**	**10.0 (n= 936)**	**24.9 (n= 1584)**
Summer	1.7 (n= 288)		35.4 (n= 288)	19.0 (n= 216)	9.5 (n= 360)	22.2 (n= 360)
Autumn	0.8 (n= 360)	7.2 (n= 360)	36.7 (n=360)	9.7 (n= 360)	13.4 (n= 288)	17.5 (n= 360)
Winter	0.2 (n= 432)	15.3 (n= 144)	34.5 (n= 504)	8.6 (n= 504)		31.4 (n= 432)
Spring	6.9 (n= 432)		27.6 (n= 432)	8.6 (n= 432)	8.3 (n=288)	26.2 (n= 432)
**2016**	**0.2 (n= 432)**		**39.6 (n= 504)**	**8.9 (n= 576)**	**12.8 (n=504)**	**14.1 (n= 432)**
Summer	0.5 (n= 216)		47.7 (n=288)	9.7 (n= 288)	4.6 (n=288)	11.6 (n= 216)
Autumn	0.0 (n= 216)		31.5 (n=216)	7.9 (n= 288)	17.5 (n= 288)	16.0 (n= 216)
**All recordings inspected**	**1.9 (n= 2016)**	**11.5 (n= 792)**	**35.2 (n= 2448)**	**12.4 (n= 2448)**	**13.1 (n= 1728)**	**23.4 (n = 2376)**

Supplementary Table S2: Information about the location and habitat classification of the six listening stations (Jackson, 2014).

**Listening Station**	**Number of deployments**	**Habitat classification (MPA Policy 2014)**	**Latitude**	**Longitude**	**Water Depth (m)**
Horn Rock	10	Shallow rocky reef	-36.25694	175.19167	18
Waiheke Island	5	Shallow sand	-36.80220	175.23470	27
Bean Rock	10	Deep mud	-36.828889	174.84722	6
Shearer Rock	10	Shallow gravel (adjacent to shallow rocky reef)	-36.61417	174.92583	21
Flat Rock	11	Deep sand (adjacent to deep rocky reef)	-36.43980	174.93140	28
Jellicoe Channel	10	Deep sand	-36.24390	174.91841	57
